# Tick-Borne Encephalitis Virus and Its European Distribution in Ticks and Endothermic Mammals

**DOI:** 10.3390/microorganisms8071065

**Published:** 2020-07-17

**Authors:** Melanie Walter, Janna R. Vogelgesang, Franz Rubel, Katharina Brugger

**Affiliations:** Unit for Veterinary Public Health and Epidemiology, University of Veterinary Medicine Vienna, 1210 Vienna, Austria; melanie.walter@vetmeduni.ac.at (M.W.); janna.vogelgesang@vetmeduni.ac.at (J.R.V.); franz.rubel@vetmeduni.ac.at (F.R.)

**Keywords:** tick-borne disease, TBE, *Ixodes ricinus*, exposure, hazard, risk, map, species distribution model, random forests

## Abstract

Tick-borne encephalitis (TBE) is the most common viral tick-borne disease in Europe causing thousands of human infections every year. Available risk maps in Europe are solely based on human incidences, but often underestimate areas with TBE virus circulation as shown by several autochthonous cases detected outside known risk areas. A dataset of more than 1300 georeferenced TBE virus detections in ticks and mammals except for humans was compiled and used to estimate the probability of TBE virus presence in Europe. For this, a random forests model was implemented using temperature- and precipitation-dependent bioclimatic variables of the WorldClim dataset, altitude, as well as land cover of the ESA GlobCover dataset. The highest probabilities of TBE virus presence were identified in Central Europe, in the south of the Nordic countries, and in the Baltic countries. The model performance was evaluated by an out-of-bag error (OOB) of 0.174 and a high area under the curve value (AUC) of 0.905. The TBE virus presence maps may subsequently be used to estimate the risk of TBE virus infections in humans and can support decision-makers to identify TBE risk areas and to encourage people to take appropriate actions against tick bites and TBE virus infections.

## 1. Introduction

In Europe, tick-borne encephalitis (TBE) is the most common viral tick-borne disease of the central nervous system. The causative agent, the TBE virus, is transmitted to humans mainly by the widespread tick species *Ixodes ricinus*, but it may also be acquired via consumption of infected unpasteurized dairy products [1]. In the European Union, around 3000 human TBE cases are reported annually [2]. It is estimated that these are one-third of all TBE virus infections, which manifested in severe clinical neurological conditions and laboratory confirmed. The other two-thirds of human TBE virus infections remain asymptomatic or have mild-clinical symptoms [2]. More detailed information on pathogenesis, clinical picture, case definition, and other interesting aspects are reviewed by Růžek et al. [3] recently.

The European Centre for Disease Prevention and Control (ECDC) provides regularly updated standardized TBE incidence maps [2]. Using the European Union TBE case definition [4], the incidence is given as numbers of cases per population of 100,000 summarized on socio-economic regions at NUTS-2 or NUTS-3 level. Another European TBE distribution map is a combination of national risk maps depicting TBE endemic regions [5]. These maps are provided by national health authorities and are based on different TBE case definitions and risk assessments (an overview for each country is given in Dobler et al. [6]). Previously, human cases were seen as an indicator for the occurrence of future infections as TBE virus circulates in certain areas over many years. Nevertheless, each year several autochthonous cases have been detected outside the official risk areas. For example, in Germany about 15% of all TBE human cases since 2001 acquired their infection outside the official risk areas [7,8].

**Figure 1:**
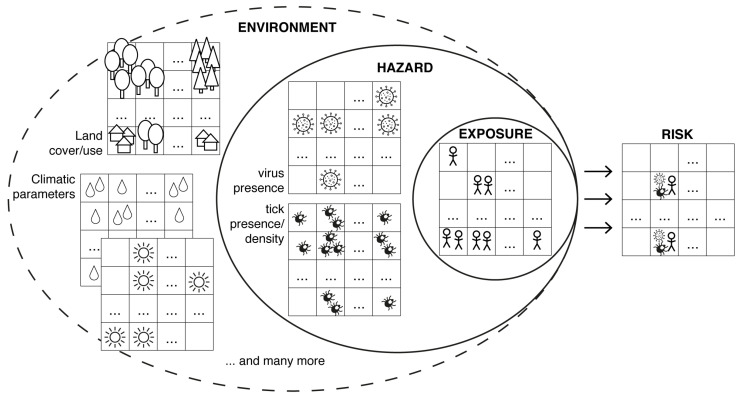
Assessing the spatial risk for human tick-borne encephalitis results from a combination of hazard (virus presence and tick presence/density) and exposure (e.g., human outdoor activity, vaccination rate) triggered by abiotic and biotic environment.

In addition to these case-based approaches, a comprehensive spatial risk assessment for human tick-borne diseases needs the identification and quantification of the hazard, i.e., the potential source of harm, and characterisation of the exposure, i.e., the intensity of contact with the hazard [9]. As depicted in Figure 1, the hazard in case of TBE is on the one hand TBE virus presence and on the other hand tick presence or density. Exposure means human occupational or recreational activities outdoors which is influenced e.g., by behavioural factors, attractiveness of the landscape, demography, or vaccination rate against TBE virus [10,11]. Here, we are focusing on the hazard of TBE virus presence. Instead of human TBE cases we compiled published TBE virus detections in both ticks as vectors and endothermic mammals as hosts. Mapping these data provides an insight into the spatial distribution of TBE virus. A so-called species distribution model (SDM) was then implemented to compile a high-resolution map of the probability of TBE virus presence. For this, the locations of TBE virus detected in ticks and endothermic mammals were used as response variables and environmental variables as predictors. SDMs have been introduced in the mid eighties [12] to estimate the potential (continental) distribution of plant or animal species depending on climate parameters [13]. In the past 15 years, SDMs have been increasingly used for predicting the potential distribution of infectious diseases as part of risk assessments [14]. For this, either the distribution of pathogens or disease cases themselves (e.g., West Nile Fever [15], Usutu virus [16], or chikungunya virus [17]) was modelled or—in the case of vector-borne pathogens—solely the vectors such as mosquitoes [18] or ticks [19]. In the case of species distribution models for TBE recent publications are focusing only on limited areas e.g., Finland [20] or the so called DACH region, i.e., Germany, Austria, Switzerland [21].

## 2. Materials and Methods

### 2.1. Tick-Borne Encephalitis Virus Detections in Ticks and Endothermic Mammals

A comprehensive literature search on TBE virus detections in ticks and endothermic mammals was conducted to compile a dataset of georeferenced locations. The search was performed on the Scopus, PubMed, and Web of Science databases for the period from the inception of these databases to September 2019. The search terms included ‘tick-borne encephalitis’, ‘tick-borne disease’, ‘TBE’, and ‘tick + encephalitis’. In addition the terms were also used in the European national languages, e.g., ‘Frühsommer-Meningoenzephalitis’ and ‘FSME’ for the German literature, ‘kleszczowe zapalenie mózgu’ for the Polish literature, ‘Fästing-buren encefalitviruset’ for Swedisch literature, or ‘encéphalite à tiques’ for the French literature. Furthermore, additional publications were added by reviewing the article references. The herein accepted virus detection methods ranged from molecular (direct) to serological (indirect) techniques and improved constantly over the past decades [22]. Although the comparability of detection methods is discussed controversially regarding the reliability of on-going TBE virus circulation [23], all of them were included in the dataset as a qualitative virus present/absent decision, which is of main interest here rather than a quantitative indication of prevalence. Locations of TBE virus detections were given as exact geographical coordinates, precise site descriptions (i.e., place and town names), or maps. In case of the latter two, the geographical coordinates were estimated or the exact coordinates were provided by the authors upon request. Focusing on the main endemic area of TBE in Europe, we concentrated on the geographical region of 2.5${}^{{}^{\circ}}$–25.0${}^{{}^{\circ}}$ E/45.0${}^{{}^{\circ}}$–62.5${}^{{}^{\circ}}$ N.

### 2.2. Environmental Variables

In this study, mapping the distribution of TBE virus in ticks and endothermic mammals included bioclimatic variables, altitude, and a classification of land cover. All variables are available with a high spatial resolution throughout Europe. The bioclimatic variables were taken from the WorldClim database version 2 [24]. Derived from high-resolution monthly temperature and precipitation data, this dataset comprises 19 bioclimatic variables such as annual mean temperature and annual precipitation. We used the dataset with a spatial resolution of 2.5 arcmin, corresponding to a mean grid cell size of about 13 km${}^{2}$. To avoid multicollinearity and known spatial artefacts [25] the variable set was reduced to 6 bioclimatic variables (Table 1). The altitude of the locations with TBE virus detections ranged from 0 to 1500 m (median 247 m).

Additionally, the satellite-based ESA GlobCover 2009 dataset was used [26]. This dataset provides 22 land cover types worldwide and 20 land cover types in the model area, respectively. Here, the types were combined into 7 main classes: (A) agriculture areas, (B) broad-leaved forest, (C) coniferous forest, (M) mixed forest, (V) low vegetation, (U) urban areas, and (X) unsuitable areas (Table 2). As the GlobCover data have a higher spatial resolution of 10 arcsec, they were aggregated to the map resolution of 2.5 arcmin by calculating modal values.

### 2.3. Random Forests Model

The random forests model is a machine-learning algorithm based on classification trees and so-called bagging methods, i.e., bootstrapping and aggregating [27]. Classification trees combine the idea of data splitting to develop a binary tree and subsequent error minimization at each knot. In a recent study, the random forests model performed best for predicting virus presence in unsampled areas compared to other species distribution models [16]. The random forests model is used to interpolate the observed locations of TBE virus presence in ticks and endothermic mammals on a regular grid indicating the probability of the TBE virus being present in a given cell. This probability ranges from 0 to 1 on a continuous scale and the traditional threshold of 0.5 was set. A value greater than 0.5 implies that TBE virus is rather present than absent. Moreover, the threshold can be interpreted as distinction between endemic and non-endemic areas for TBE virus.

Besides environmental predictors, the random forests model also needs presence and (pseudo-)absence grid cells of the response variable. A grid cell is defined as a presence cell, if at least one TBE virus-positive tick or endothermic mammal was found in it. As absence grid cells were usually not available, they were generated synthetically. Grid cells with virus absence were randomly selected with numbers equal to grid cells with virus presence, but at least 12 grid cells away from a presence cell. The selection of absence grid cells has an impact on model performance [28], so we ran the model 1000 times in order to receive a representative average. Further, the model was tuned using a number of 1000 trees and an inflation of 1.5 of the variable number at each step.

The out-of-bag (OOB) error was used for quantifying the model prediction error. This measure utilizes bootstrapping and aggregating to sub-sample the observation data into training samples. Then the mean prediction error of each sample, using only the trees that did not have it in their bootstrap sample, is calculated [27]. Additionally, the model performance was evaluated by calculating the threshold-independent area under the receiver operated curve (AUC) value, defined within the range of 0.5 and 1.0, where 0.5 implies totally random predictions [29].

The implementation of the random forests model and all calculations were conducted using the open-source statistical software R [30]. The package raster was used for compiling the gridded datasets and mapping the results [31], the package randomForest for implementing the random forests model [32], and the package spThin for spatially thinning clustered TBE virus locations [33].

## 3. Results

**Figure 2:**
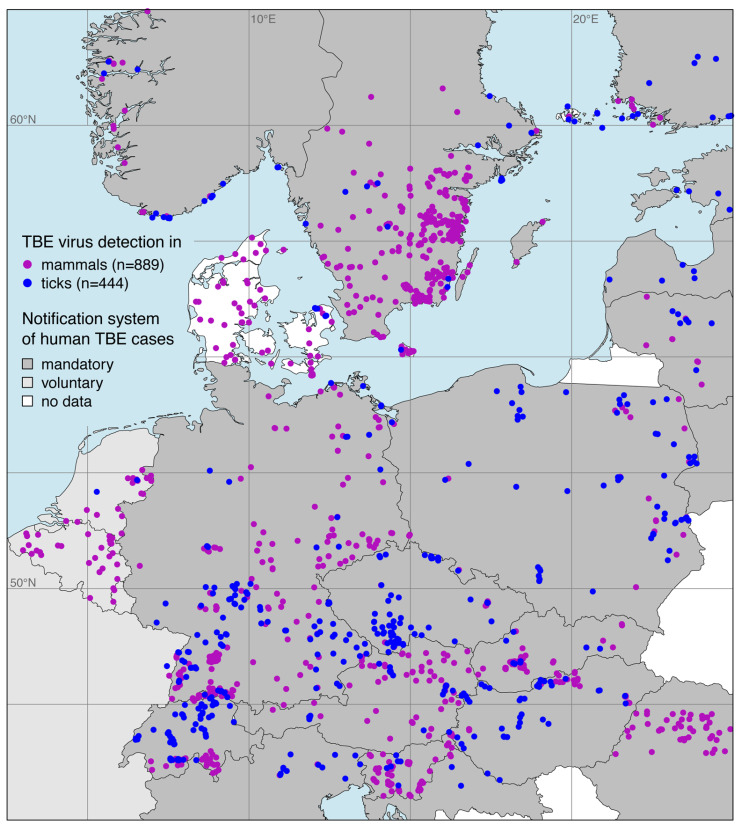
Georeferenced tick-borne encephalitis (TBE) virus detections in ticks (blue), mainly the castor bean tick *Ixodes ricinus*, and endothermic mammals except for humans (purple). Additionally, the sort of national notification system for human TBE cases is given.

The georeferenced locations of TBE virus detections in both ticks and endothermic mammals exept for humans are shown in Figure 2. Focusing on the main endemic area in Europe, 889 locations with TBE virus detections in endothermic mammals and 444 locations with detections in ticks were taken from of a total of 133 publications. The complete list is given in the Supplementary Materials.

**Figure 3:**
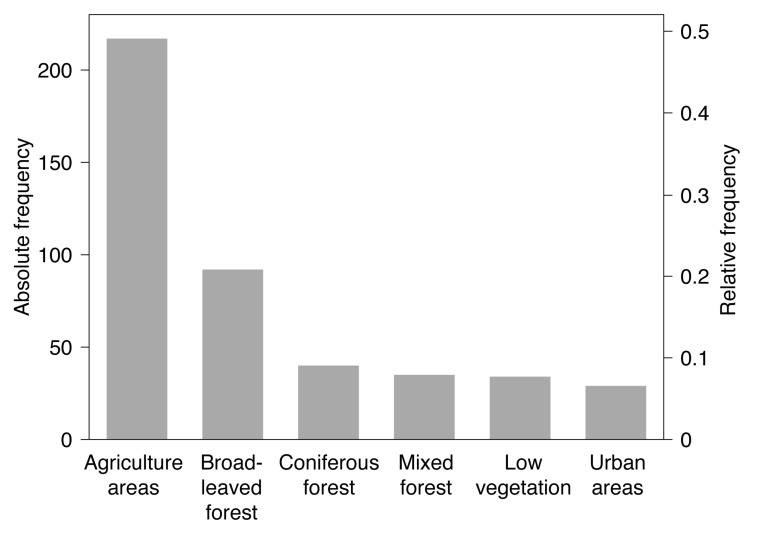
Frequency distribution of locations with tick-borne encephalitis virus detections in both ticks and endothermic mammals of the subsample used for the random forests model (n=447). Absolute frequencies depict the number of locations, relative frequencies the fraction of locations in the different land cover classes.

Georeferenced detections of TBE virus-infected ticks were mostly *Ixodes ricinus* (n=350, 78.8%). Virus detections in endothermic mammals include wild mammals such as rodents, deer, or wild boars (n=372, 41.8%) as well as domestic animals such as dogs, horses, or cattle (n=517, 58.2%). Please note that at some locations TBE virus was detected in more than one species. By using a spatial thinning algorithm, the dataset was optimized to address the known problems with spatial sampling biases, e.g., due to clustered data [34]. With an appropriate distance of 20 km the dataset was reduced to 447 (35.5%) of previously 1333 locations where TBE virus was detected. As depicted in Figure 3, the majority of the selected locations lay in the land cover class agriculture areas (n=217, 48.5%), followed by broad-leaved forest (n=92, 20.6%) and coniferous forest (n=40, 8.9%).

**Figure 4:**
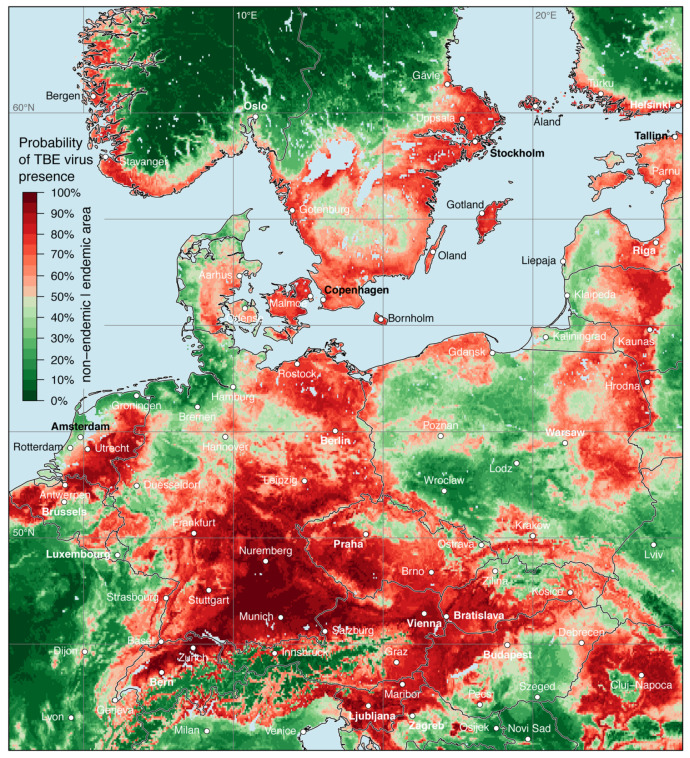
Probability of tick-borne encephalitis virus presence in both ticks and endothermic mammals except for humans. Endemic areas, i.e., a probability from 0.5 to 1.0, are indicated in light red to dark red and non-endemic areas are indicted from dark green to light green.

The result of the random forests model, i.e., the distribution map of the probability of TBE virus presence in Europe is shown in Figure 4. As discussed above, the spatial probability of TBE virus presence was predicted based on virus detections and environmental predictors. A probability between 0.5 and 1.0 indicates that an area (grid cell) is more likely to be categorized as a virus presence/endemic cell than an absence/non-endemic cell by the random forests model. On the other hand, a probability lower than 0.5 indicates an area, that is more likely to be categorized as an absence than a presence cell.

For several regions a high probability of TBE virus presence was identified and TBE virus circulation can reasonably be assumed. Starting from the western part of the model area, a high TBE virus presence was predicted for the eastern part of France (mainly Alsace), Belgium except northern Wallonia, the Netherlands, and Denmark. Only along the coastal areas of the North Sea the probability was lower. Major parts of Germany were indicated as TBE virus-endemic area, with highest probabilities determined for Bavaria and Baden-Württemberg. The western part of the federal state Schleswig-Holstein, East Frisian Peninsula, and Oldenburg Münsterland in Lower Saxony, as well as the Ruhr region and Münsterland in North Rhine-Westphalia were indicated as non-endemic areas.

Apart from the Alpine region, high probabilities of TBE virus presence were predicted for almost all of Switzerland, Austria, Slovenia, and the northern part of Italy along the foothills. High probabilities were also calculated for large parts of the Czech Republic, Slovakia except the Western Carpathians, the northern parts of Hungary, along the eastern and western border of Poland, and in the Baltic countries. In the northern part of the model area, high probabilities were predicted for the south-western coast of Norway, in Sweden south of the biogeographically boarder traditionally named *Limes norrlandicus*, the southern coast of Finland, and the Åland Islands.

The model performance was evaluated by an out-of-bag error of OOB = 0.174, that means only 17.4% of all OOB observations (not used for fitting a specific tree) were classified incorrectly by the random forests model, but 82.6% were classified correctly. Further, a verification measure of AUC = 0.905 indicates a good model performance as well as a sensitivity of 0.842 and a specificity of 0.813.

**Figure 5:**
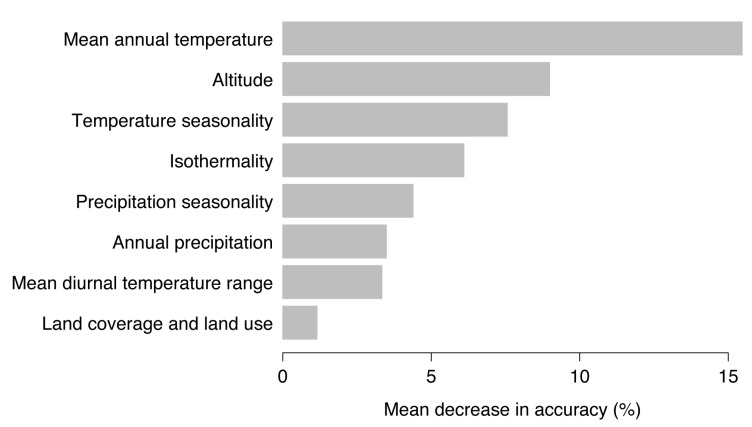
Ranking of the eight used environmental variables according to the mean decrease in accuracy.

Random forests models provide a ranking of the most important environmental variables for model fitting and prediction by means of decrease in accuracy. High values of mean decrease in accuracy indicate variables that are important for model performance [35]. As shown in Figure 5, mean annual temperature, altitude, and temperature seasonality were most important to predict the probability of TBE virus presence. Removing the mean annual temperature from the set of possible environmental predictors would result in a mean decrease in model accuracy of 15.5%, for altitude of 9.0%, and temperature seasonality of 7.6%.

## 4. Discussion

Here, we present a newly compiled dataset of georeferenced TBE virus detections in ticks and endothermic mammals. This dataset together with environmental variables was used for mapping the probability of TBE virus presence in different parts of Europe. For this a random forests model, a machine-learning species distribution model, was implemented.

Species distribution models represent an alternative approach for predicting the potential geographical distribution of infectious diseases by means of georeferenced pathogen and/or disease detections and associated environmental or disease-influencing variables. Such models treat the entire disease system, i.e., in case of TBE the virus–vector–host interaction, as a so-called black box. As an advantage of this approach, SDMs are relatively easy and cost-effective to implement and deliver results quickly [13]. One limitation is that a lack of data on TBE virus detections in some regions can lead to false negative results (i.e., low probability of virus presence vs. on-going TBE virus circulation) [13].

For some years, there is a trend away from case-based towards hazard/exposure-based risk assessments for tick-borne diseases such as TBE or Lyme borreliosis [36,37]. Complementary risk assessments are needed to encourage the local population in endemic regions but also travellers to apply proper prophylactic measures to avoid tick bites and tick-borne infections. Available incidence maps of human TBE cases and maps of national risk areas have usually a rather coarse spatial resolution. As based merely on proven human TBE cases, they may underestimate the risk in areas outside the known risk areas.

In addition, using virus detections in ticks and endothermic mammals as a response variable has several advantages. Probably the most important point is that georeferenced locations of virus detections in ticks and endothermic mammals are usually published in scientific literature. In contrast, human disease data are so-called sensitive personal data as specified in data protection regulations [38]. For nations with mandatory disease notification systems (Figure 2), human incidence data are only provided on NUT-2 or NUT-3 level, which is far too coarse for SDMs. Apart from that, the sampled mammal species are site-loyal and have a home range from 0.002 km${}^{2}$ in case of the bank vole (*Myodes glareolus*[39]) or up to approximately 0.23 km${}^{2}$ in case of roe deer (*Capreolus capreolus*[40]). Possible inaccuracies of the georeferenced locations were compensated by the choice of the model resolution of 2.5 arcmin, which correspond to a mean grid cell size of 13 km${}^{2}$.

A visual comparison of the here presented map of the probability of TBE virus presence in different areas in Europe with the two so far available human risk maps [2,5] shows good match with known human risk areas (e.g., Bavaria and Baden-Württemberg in Germany, Slovenia, western Hungary, southern Czech Republic, and western Slovakia). Additionally, areas with a moderate probability of virus presence were indicated in so far non-official risk areas, but with reported autochthonous human cases (e.g., northern federal states of Germany [7], the Netherlands [41]). Furthermore, directly comparing the mean probability of TBE virus presence with the mean human TBE incidence (numbers of cases per population of 100,000) for the period 2001–2019 [8] in Germany on NUTS-3 level results in a strong Spearman’s correlation coefficient of 0.689 (p<0.001).

A high probability of TBE virus presence is not always associated with a high human TBE incidence. One reason for this is the non-homogenous human population density in Europe. For instance, in southern Sweden there is a high probability of virus presence, but human cases are mainly reported in areas with a high human population density, e.g., in the greater Stockholm region and in the last years increasingly around the great lakes Vättern, Vänern, and Mälaren [42]. A similar pattern is also observed in northern Germany where in sparsely populated regions only individual human TBE cases were reported [8]. Another reason is the vaccination coverage which varies widely between various regions in Europe [43]. For example, Austria population has a vaccination coverage of more than 80% (fraction of the population which received at least one TBE vaccination, i.e., ≥1 vaccine dose) since the end of the nineties and this has led to a significant decline in the annual number of human TBE cases [44,45].

The so far available (national) risk maps are based on various human TBE case definitions and risk assessments [46], and thus comparisons between different parts of Europe should be treated with caution. In contrast, for the map of TBE virus presence compiled here a uniform cross-national approach was applied and provides for the first time an Europewide consistent insight on the presence of TBE virus. The next step for the identification and quantification of a hazard will be the simulation of the density of host-seeking ticks European wide as already available for some European countries [47,48]. Together with the characterisation of the human exposure, this is necessary towards a comprehensive spatial risk assessment.

## Figures and Tables

**Table 1:** Median and range of bioclimatic variables observed at the locations of tick-borne encephalitis virus detections in both ticks and endothermic mammals.

Bioclimatic Variables	Abbr.	Min.	Median	Max.	Unit
Mean annual temperature	bio01	2.9	8.3	13.0	°C
Mean diurnal temperature range	bio02	4.7	8.0	11.2	°C
Isothermality	bio03	20.9	29.7	36.0	°C
Temperature seasonality	bio04	488.5	701.6	868.4	°C
Annual precipitation	bio12	483.0	710.0	3451.0	mm/a
Precipitation seasonality	bio15	8.8	26.6	49.3	mm/a

**Table 2:** GlobCover land cover types [26] occurring in the model area were combined into 7 main classes: (A) agriculture areas, (B) broad-leaved forest, (C) coniferous forest, (M) mixed forest, (V) low vegetation, (U) urban areas, and (X) unsuitable areas.

Class	GlobCover Global Legend	Value
(A)	Post-flooding or irrigated croplands	11
	Rainfed croplands	14
	Mosaic cropland (50–70%)/vegetation (grassland, shrubland, forest) (20–50%)	20
	Mosaic vegetation (grassland, shrubland, forest) (50–70%)/cropland (20–50%)	30
(B)	Closed to open (>15%) broadleaved evergreen and/or semi-deciduous forest (>5 m)	40
	Closed (>40%) broadleaved deciduous forest (>5 m)	50
	Open (15–40%) broadleaved deciduous forest (>5 m)	60
(C)	Closed (>40%) needleleaved evergreen forest (>5 m)	70
	Open (15–40%) needleleaved deciduous or evergreen forest (>5 m)	90
(M)	Closed to open (>15%) mixed broadleaved and needleleaved forest (>5 m)	100
	Mosaic forest/shrubland (50–70%)/grassland (20–50%)	110
(V)	Mosaic grassland (50–70%)/forest/shrubland (20–50%)	120
	Closed to open (>15%) shrubland (<5 m)	130
	Closed to open (>15%) grassland	140
	Sparse (>15%) vegetation (woody vegetation, shrubs, grassland)	150
	Closed to open (>15%) vegetation (grassland, shrubland, woody vegetation) on regularly flooded or waterlogged soil – Fresh, brackish or saline water	180
(U)	Artificial surfaces and associated areas (urban areas >50%)	190
(X)	Bare areas	200
	Water bodies	210
	Permanent snow and ice	220

## Supplementary Materials

The following are available online at https://www.mdpi.com/2076-2607/8/7/1065/s1. Complete list of the references with georeferenced tick-borne encephalitis virus detections in ticks and endothermic mammals except for humans used in this study.
