# Supplementary material for: Tick-Borne Encephalitis Virus and Its European Distribution in Ticks and Endothermic Mammals
Source: Microorganisms. 2020 Jul 17;8(7):1065. doi: 10.3390/microorganisms8071065 (PMC7409098; doi:10.3390/microorganisms8071065)
Supplement: Supplementary file 1 [file microorganisms-08-01065-s001.pdf]

Appendix A

Complete list of the references with georeferenced tick-borne encephalitis virus detections in ticks and endothermic mammals except for humans used in this study.

Country	References	Country	References
Austria	Duscher et al. (2015)	Germany	Bestehorn et al. (2018)
	Ecker et al. (1999)		Bingsohn et al. (2013)
	Kunz et al. (1967)		Böhm et al. (2017)
	Kunz et al. (1969)		Boelke et al. (2019)
	Radda et al. (1971)		Bröker et al. (2019)
	Weissenböck et al. (1998)		Dobler (2018)
Belgium	Roelandt et al. (2011)		Ecker et al. (1999)
	Roelandt et al. (2014)		Eistetter et al. (1998)
	Roelandt et al. (2016)		Frimmel et al. (2010)
	Tavernier et al. (2015)		Frimmel et al. (2014)
Czech Republic	Danielová et al. (2002)		Frimmel et al. (2016)
	Danielová et al. (2008)		Gerth et al. (1995)
	Ecker et al. (1999)		Holbach and Oehme (2002)
	Ernek et al. (1967)		Janitza-Futterer (2003)
	Grešíková and Nosek (1967)		Kiffner et al. (2011)
	Hönig et al. (2015)		Klaus et al. (2010a)
	Kožuch et al. (1967)		Klaus et al. (2010b)
	Krivanec et al. (1988)		Klaus et al. (2012)
	Nosek et al. (1970)		Klaus et al. (2013)
	Širmarová et al. (2014)		Kupča et al. (2010)
	Weidmann et al. (2011)		Mehlhorn et al. (2015)
Denmark	Agergaard et al. (2019)		Müller (1970)
	Andersen et al. (2019b)		Müller (2006)
	Andersen et al. (2019a)		Oehme et al. (2002)
	Fomsgaard et al. (2013)		Pluta (2011)
	Lindhe et al. (2009)		Ramelow et al. (1993)
	Petersen et al. (2019)		Rieger et al. (1999)
	Skarphédinsson et al. (2005)		Skuballa (2011)
Estonia	Katargina et al. (2013)		Stefanoff et al. (2012)
Finland	Brummer-Korvenkontio et al. (1973)		Süss et al. (2006)
	Ecker et al. (1999)	Hungary	Süss et al. (1997)
	Laaksonen et al. (2017)		Ecker et al. (1999)
	Levanov et al. (2016)		Lakos et al. (2019)
	Lindblom et al. (2014)		Nosek et al. (1970)
	Smura et al. (2019)		Pintér et al. (2013)
	Sormunen et al. (2018)		Šikutová et al. (2009)
France	Tonteri et al. (2016)	Italy	Zöldi et al. (2014)
	Bestehorn et al. (2018)		Cagnacci et al. (2012)
	Ecker et al. (1999)		Capelli et al. (2012)
Germany	Perez-Eid et al. (1992)		Carpi et al. (2009)
	Achazi et al. (2011)		Carpi et al. (2009)
	Balling et al. (2014)		D'Agaro et al. (2009)
	Balling et al. (2015)		Hudson et al. (2001)

Country	References	Country	References
Liechtenstein	Rosà et al. (2019)	Poland	Stefanoff et al. (2012)
Lithuania	Aberham et al. (1992)		Wójcik-Fatla et al. (2011)
	Han et al. (2005)		Zając et al. (2017)
	Juceviciene et al. (2005)	Romania	Salat et al. (2017)
Netherlands	Katargina et al. (2013)	Slovakia	Cagnacci et al. (2012)
	Jahfari et al. (2017)		Csank et al. (2018)
Norway	Rijks et al. (2019)		Labuda et al. (2002)
	Andreassen et al. (2012)		Nosek et al. (1970)
	Csángó et al. (2004)	Slovenia	Durmiši et al. (2011)
	Kjelland et al. (2018)		Knap et al. (2012)
	Larsen et al. (2014)	Sweden	Asghar et al. (2017)
	Paulsen et al. (2018)		Brinck et al. (1967)
	Sidorenko et al. (2018)		Brinkley et al. (2008)
	Traavik (1973)		Grandi et al. (2018)
	Traavik et al. (1978)		Lindblom et al. (2014)
Poland	Ytrehus et al. (2013)		Melik et al. (2007)
	Bajer et al. (2014)		Pettersson et al. (2014)
	Biernat et al. (2014b)		von Zeipel et al. (1959)
	Biernat et al. (2014a)		von Zeipel (1959)
	Biernat et al. (2016)	Switzerland	Burri et al. (2011)
	Cisak et al. (2002)		Casati (2006)
	Cisak et al. (2012)		Casati Pagani et al. (2019)
Poland	Cuber et al. (2015)		Ecker et al. (1999)
	Danielová et al. (2010)		Gäumann et al. (2010)
	Drelich et al. (2014)		Ineichen (2019)
	Grzybek et al. (2018)		Lommano et al. (2012)
	Katargina et al. (2013)		Rieille et al. (2014)
	Kowalewska et al. (1997)		Rieille et al. (2017)
	Makówka et al. (2009)		Wandeler et al. (1972)
	Mierzejewska et al. (2015)		Wicki et al. (2000)
	Stefanoff et al. (2008)		

References

Aberham C, Radda A, Holzmann H, Krech R (1992) Detection of tick-borne encephalitis (TBE) virus in Liechtenstein. Zbl Bakteriol 277(4):554–560, DOI 10.1016/S0934-8840(11)80481-0

Achazi K, Růžek D, Donoso-Mantke O, Schlegel M, Ali HS, Wenk M, Schmidt-Chanasit J, Ohlmeyer L, Rühe F, Vor T, Kiffner C, Kallies R, Ulrich RG, Niedrig M (2011) Rodents as sentinels for the prevalence of tick-borne encephalitis virus. Vector Borne Zoonotic Dis 11(6):641–647, DOI 10.1089/vbz.2010.0236

Agergaard CN, Rosenstierne MW, Bødker R, Rasmussen M, Andersen PHS, Fomsgaard (2019) New tick-borne encephalitis virus hot spot in Northern Zealand, Denmark, October 2019. Euro Surveill 24(43):pii=1900639, DOI 10.2807/1560-7917.es.2019.24.43.1900639

Andersen NS, Bestehorn M, Chitimia-Dobler L, Kolmos HJ, Jensen PM, Dobler G, Skarphédinsson S (2019a) Phylogenetic characterization of tick-borne encephalitis virus from Bornholm, Denmark. Ticks Tick Borne Dis 10(3):533–539, DOI 10.1016/j.ttbdis.2018.12.008

Andersen NS, Larsen SL, Olesen CR, Stiasny K, Kolmos HJ, Jensen PM, Skarphédinsson S (2019b) Continued expansion of tick-borne pathogens: Tick-borne encephalitis virus complex and *Anaplasma phagocytophilum* in Denmark. Ticks Tick Borne Dis 10(1):115–123, DOI 10.1016/j.ttbdis.2018.09.007

Andreassen A, Jore S, Cuber P, Dudman S, Tengs T, Isaksen K, Hygen HO, Viljugrein H, Ånestad G, Ottesen P, Vainio K (2012) Prevalence of tick borne encephalitis virus in tick nymphs in relation to climatic factors on the southern coast of Norway. Parasit Vectors 5(1):177, DOI 10.1186/1756-3305-5-177

Asghar N, Pettersson JHO, Dinnetz P, Andreassen Å, Johansson M (2017) Deep sequencing analysis of tick-borne encephalitis virus from questing ticks at natural foci reveals similarities between quasispecies pools of the virus. J Gen Virol 98(3):413–421, DOI 10.1099/jgv.0.000704

Bajer A, Mierzejewska EJ, Rodo A, Bednarska M, Kowalec M, Welc-Falęciak R (2014) The risk of vector-borne infections in sled dogs associated with existing and new endemic areas in Poland. Vet Parasitol 202(3–4):276–286, DOI 10.1016/j.vetpar.2013.12.033

Balling A, Plessow U, Beer M, Pfeffer M (2014) Prevalence of antibodies against tick-borne encephalitis virus in wild game from Saxony, Germany. Ticks Tick Borne Dis 5(6):805–809, DOI 10.1016/j.ttbdis.2014.06.007

Balling A, Beer M, Gniel D, Pfeffer M (2015) [Prevalence of antibodies against tick-borne encephalitis virus in dogs from Saxony, Germany]. Berl Munch Tierarztl Wochenschr 128(7/8):297–303, DOI 10.2376/0005-9366-128-297, in German

Bestehorn M, Weigold S, Kern WV, Chitimia-Dobler L, Mackenstedt U, Dobler G, Borde JP (2018) Phylogenetics of tick-borne encephalitis virus in endemic foci in the upper Rhine region in France and Germany. PLoS ONE 13(10):e0204790, DOI 10.1371/journal.pone.0204790

Biernat B, Cieniuch S, Stańczak J (2014a) Detection of TBEV RNA in *Ixodes ricinus* ticks in north-eastern Poland. Ann Agric Environ Med 21(4):689–692, DOI 10.5604/12321966.1129915

Biernat B, Karbowiak G, Werszko J, Stańczak J (2014b) Prevalence of tick-borne encephalitis virus (TBEV) RNA in *Dermacentor reticulatus* ticks from natural and urban environment, Poland. Exp Appl Acarol 64(4):543–551, DOI 10.1007/s10493-014-9836-5

Biernat B, Karbowiak G, Stańczak J, Masny A, Werszko J (2016) The first detection of the tick-borne encephalitis virus (TBEV) RNA in *Dermacentor reticulatus* ticks collected from the lowland European bison (*Bison bonasus bonasus* L.). Acta Parasitol 61(1):130–135, DOI 10.1515/ap-2016-0017

Bingsohn L, Beckert A, Zehner R, Kuch U, Oehme R, Kraiczy P, Amendt J (2013) Prevalences of tick-borne encephalitis virus and Borrelia burgdorferi sensu lato in *Ixodes ricinus* populations of the Rhine-Main region, Germany. Ticks Tick Borne Dis 4(3):207–213, DOI 10.1016/j.ttbdis.2012.11.012

Boelke M, Bestehorn M, Marchwald B, Kubinski M, Liebig K, Glanz J, Schulz C, Dobler G, Monazahian M, Becker SC (2019) First isolation and phylogenetic analyses of tick-borne encephalitis virus in Lower Saxony, Germany. Viruses 11(5):462, DOI 10.3390/v11050462

Böhm B, Schade B, Bauer B, Hoffmann B, Hoffmann D, Ziegler U, Beer M, Klaus C, Weissenböck H, Böttcher J (2017) Tick-borne encephalitis in a naturally infected sheep. BMC Vet Res 13(1):267, DOI 10.1186/s12917-017-1192-3

Brinck P, Johnels A, Lundholm B, Svedmyr A, von Zeipel G, Zetterberg B (1967) Small mammals in Sweden as hosts of tick-borne encephalitis virus and vagrant ectoparasites. Oikos 18(1):124, DOI 10.2307/3564640

Brinkley C, Nolskog P, Golovljova I, Lundkvist Å, Bergström T (2008) Tick-borne encephalitis virus natural foci emerge in western Sweden. Int J Med Microbiol 298:73–80, DOI 10.1016/j.ijmm.2007.12.005

Bröker M, Chitimia-Dobler L, Dobler G (2019) [Ticks and tick-borne encephalitis in the district Marburg-Biedenkopf]. Philippia 17(4):279–288, in German

Brummer-Korvenkontio M, Saikku P, Korhonen P, Oker-Blom N (1973) Arboviruses in Finland. I. Isolation of tick-borne encephalitis (TBE) virus from arthropods, vertebrates, and patients. Am J Trop Med Hyg 22(3):382–389

Burri C, Bastic V, Maeder G, Patalas E, Gern L (2011) Microclimate and the zoonotic cycle of tick-borne encephalitis virus in Switzerland. J Med Entomol 48(3):615–627, DOI 10.1603/me10180

Cagnacci F, Bolzoni L, Rosà R, Carpi G, Hauffe H, Valent M, Tagliapietra V, Kazimirova M, Koci J, Stanko M, Lukan M, Henttonen H, Rizzoli A (2012) Effects of deer density on tick infestation of rodents and the hazard of tick-borne encephalitis. I: Empirical assessment. Int J Parasitol 42(4):365–372, DOI 10.1016/j.ijpara.2012.02.012

Capelli G, Ravagnan S, Montarsi F, Ciocchetta S, Cazzin S, Porcellato E, Babiker AM, Cassini R, Salviato A, Cattoli G, Otranto D (2012) Occurrence and identification of risk areas of *Ixodes ricinus*-borne pathogens: a cost-effectiveness analysis in north-eastern Italy. Parasit Vectors 5(1):61, DOI 10.1186/1756-3305-5-61

Carpi G, Bertolotti L, Rosati S, Rizzoli A (2009) Prevalence and genetic variability of tick-borne encephalitis virus in host-seeking *Ixodes ricinus* in northern Italy. J Gen Virol 90(12):2877–2883, DOI 10.1099/vir.0.013367-0

Casati S (2006) Diversity of the population of tick-borne encephalitis virus infecting *Ixodes ricinus* ticks in an endemic area of central Switzerland (Canton Bern). J Gen Virol 87(8):2235–2241, DOI 10.1099/vir.0.81783-0

Casati Pagani S, Frigerio Malossa S, Klaus C, Hoffmann D, Beretta O, Bomio-Pacciorini N, Lazzaro M, Merlani G, Ackermann R, Beuret C (2019) First detection of TBE virus in ticks and sero-reactivity in goats in a non-endemic region in the southern part of Switzerland (Canton of Ticino). Ticks Tick Borne Dis 10(4):868–874, DOI 10.1016/j.ttbdis.2019.04.006

Cisak E, Chmielewska-Badora J, Rajtar B, Dutkiewicz J (2002) Study on the occurrence of *Borrelia burgdorferi* sensu lato and tick-borne encephalitis virus (TBEV) in ticks collected in Lublin region (eastern Poland). Ann Agric Environ Med 9(1):105–110

Cisak E, Wójcik-Fatla A, Sroka J, Zając V, Bilska-Zając E, Chmurzyńska E, Dutkiewicz J (2012) Prevalence of tick-borne encephalitis virus antibodies in domestic and game animals from Eastern Poland. Bull Vet Inst Pulawy 56(3):275–278, DOI 10.2478/v10213-012-0049-6

Csángó PA, Blakstad E, Kirtz GC, Pedersen JE, Czettel B (2004) Tick-borne encephalitis in Southern Norway. Emerg Infect Dis 10(3):533–534, DOI 10.3201/eid1003.020734

Csank T, Drzewniocková P, Korytár L, Major P, Gyuranecz M, Pistl J, Bakonyi T (2018) A serosurvey of flavivirus infection in horses and birds in Slovakia. Vector Borne Zoonotic Dis 18(4):206–213, DOI 10.1089/vbz.2017.2216

Cuber P, Andreassen Å, Vainio K, Asman M, Dudman S, Szilman P, Szilman E, Ottesen P, Ånestad G, Cieśla-Nobis S, Solarz K (2015) Risk of exposure to ticks (Ixodidae) and the prevalence of tick-borne encephalitis virus (TBEV) in ticks in Southern Poland. Ticks Tick Borne Dis 6(3):356–363, DOI 10.1016/j.ttbdis.2015.02.010

D'Agaro P, Martinelli E, Burgnich P, Nazzi F, Fabbro SD, Iob A, Ruscio M, Pischiutti P, Campello C (2009) Prevalence of tick-borne encephalitis virus in *Ixodes ricinus* from a novel endemic area of North Eastern Italy. J Med Virol 81(2):309–316, DOI 10.1002/jmv.21389

Danielová V, Holubová J, Daniel M (2002) Tick-borne encephalitis virus prevalence in *Ixodes ricinus* ticks collected in high risk habitats of the South-Bohemian region of the Czech Republic. Exp Appl Acarol 26(1/2):145–151, DOI 10.1023/a:1020966605960

Danielová V, Schwarzová L, Materna J, Daniel M, Metelka L, Holubová J, Kříž B (2008) Tick-borne encephalitis virus expansion to higher altitudes correlated with climate warming. Int J Med Microbiol 298(S1):68–72, DOI 10.1016/j.ijmm.2008.02.005

Danielová V, Daniel M, Schwarzová L, Materna J, Rudenko N, Golovchenko M, Holubová J, Grubhoffer L, Kilián P (2010) Integration of a tick-borne encephalitis virus and *Borrelia burgdorferi* sensu lato into mountain ecosystems, following a shift in the altitudinal limit of distribution of their vector, *Ixodes ricinus* (Krkonoše Mountains, Czech Republic). Vector Borne Zoonotic Dis 10(3):223–230, DOI 10.1089/vbz.2009.0020

Dobler G (2018) Personal communication.

Drelich A, Andreassen Å, Vainio K, Kruszyński P, Wąsik TJ (2014) Prevalence of tick-borne encephalitis virus in a highly urbanized and low risk area in Southern Poland. Ticks Tick Borne Dis 5(6):663–667, DOI 10.1016/j.ttbdis.2014.04.020

Durmiši E, Knap N, Saksida A, Trilar T, Duh D, Avšič-Županc T (2011) Prevalence and molecular characterization of tick-borne encephalitis virus in *Ixodes ricinus* ticks collected in Slovenia. Vector Borne Zoonotic Dis 11(6):659–664, DOI 10.1089/vbz.2010.0054

Duscher GG, Wetscher M, Baumgartner R, Walder G (2015) Roe deer sera used for TBE surveillance in Austria. Ticks Tick Borne Dis 6(4):489–493, DOI 10.1016/j.ttbdis.2015.03.018

Ecker M, Allison SL, Meixner T, Heinz FX (1999) Sequence analysis and genetic classification of tick-borne encephalitis viruses from Europe and Asia. J Gen Virol 80(1):179–185, DOI 10.1099/0022-1317-80-1-179

Eistetter S, Kimming P, Oehme R (1998) [Untersuchungen zur Epidemiologie der FSME in Baden-Württemberg: Bestimmung der Zeckeninfestaionsrate mithilfe eines modifizierten PCR-Verfahrens]. Bundesgesundheitsbla 41:62–66, in German

Ernek E, Kožuch O, Grešíková M (1967) The distribution of antibodies against tick-borne encephalitis virus in domestic animals in the Tribeč region. Bull World Health Organ 36(Suppl. 1):73–80

Fomsgaard A, Fertner ME, Essbauer S, Nielsen AY, Frey S, Lindblom P, Lindgren PE, Bødker R, Weidmann M, Dobler G (2013) Tick-borne encephalitis virus, Zealand, Denmark, 2011. Emerg Infect Dis 19(7):1171–1173, DOI 10.3201/eid1907.130092

Frimmel S, Krienke A, Riebold D, Löbermann M, Littmann M, Fiedler K, Klaus C, Süss J, Reisinger E (2010) Tick-borne encephalitis virus in humans and ticks in Northeastern Germany. Deut Med

Wochenschr 135(27):1393–1396, DOI 10.1055/s-0030-1262424

Frimmel S, Krienke A, Riebold D, Loebermann M, Littmann M, Fiedler K, Klaus C, Süss J, Reisinger EC (2014) Tick-borne encephalitis virus habitats in North East Germany: reemergence of TBEV in ticks after 15 years of inactivity. Biomed Res Int 2014:1–5, DOI 10.1155/2014/308371

Frimmel S, Leister M, Löbermann M, Feldhusen F, Seelmann M, Süss J, Reisinger EC (2016) Seroprevalence of tick-borne-encephalitis virus in wild game in Mecklenburg-Western Pomerania (north-eastern Germany). Ticks Tick Borne Dis 7(6):1151–1154, DOI 10.1016/j.ttbdis.2016.08.004

Gäumann R, Muhlemann K, Strasser M, Beuret CM (2010) High-throughput procedure for tick surveys of tick-borne encephalitis virus and its application in a national surveillance study in Switzerland. Appl Environ Microbiol 76(13):4241–4249, DOI 10.1128/aem.00391-10

Gerth HJ, Grimshandl D, Stage B, Döller G, Kunz C (1995) Roe deer as sentinels for endemicity of tick-borne encephalitis virus. Epidemiol Infect 115(2):355–365, DOI 10.1017/s0950268800058477

Grandi G, Aspán A, Pihl J, Gustafsson K, m FE, Jinnerot T, derlund RS, Chirico J (2018) Detection of tick-borne pathogens in lambs undergoing prophylactic treatment against ticks on two Swedish farms. Front Vet Sci 5:72, DOI 10.3389/fvets.2018.00072

Grešíková M, Nosek J (1967) Isolation of tick-borne encephalitis virus from *Ixodes ricinus* ticks in the Tribeč region. Bull World Health Organ 36(Suppl. 1):67–71

Grzybek M, Alsarraf M, Tołkacz K, Behnke-Borowczyk J, Biernat B, Stańczak J, Strachecka A, Guz L, Szczepaniak K, Paleolog J, Behnke JM, Bajer A (2018) Seroprevalence of TBEV in bank voles from Poland—a long-term approach. Emerg Microbes Infect 7(1):1–8, DOI 10.1038/s41426-018-0149-3

Han X, Juceviciene A, Uzcategui NY, Brummer-Korvenkontio H, Zygutiene M, Jääskeläinen A, Leinikki P, Vapalahti O (2005) Molecular epidemiology of tick-borne encephalitis virus in *Ixodes ricinus* ticks in Lithuania. J Med Virol 77(2):249–256, DOI 10.1002/jmv.20444

Holbach M, Oehme R (2002) [TBE and Lyme borreliosis. Spread of pathogens and risk of illness in a TBE region]. MMW Fortschr Med 144(Suppl. 4):113–118, in German

Hönig V, Svec P, Halas P, Vavruskova Z, Tykalova H, Kilian P, Vetiskova V, Dornakova V, Sterbova J, Simonova Z, Erhart J, Sterba J, Golovchenko M, Rudenko N, Grubhoffer L (2015) Ticks and tick-borne pathogens in South Bohemia (Czech Republic) – Spatial variability in *Ixodes ricinus* abundance, *Borrelia burgdorferi* and tick-borne encephalitis virus prevalence. Ticks Tick Borne Dis 6(5):559–567, DOI 10.1016/j.ttbdis.2015.04.010

Hudson PJ, Rizzoli A, Rosa R, Chemini C, Jones LD, Gould EA (2001) Tick-borne encephalitis virus in northern Italy: molecular analysis, relationships with density and seasonal dynamics of *Ixodes ricinus*. Med Vet Entomol 15(3):304–313, DOI 10.1046/j.0269-283x.2001.00317.x

Ineichen F (2019) Personal communication.

Jahfari S, de Vries A, Rijks JM, Van Gucht S, Vennema H, Sprong H, Rockx B (2017) Tick-borne encephalitis virus in ticks and roe deer, the Netherlands. Emerg Infect Dis 23(6):1028–1030, DOI 10.3201/eid2306.161247

Janitza-Futterer D (2003) [Seroepidemiological investigation of endemic TBE infection in a horse and dog population in southwestern Germany]. PhD thesis, Ludwig Maximilian University of Munich, Germany, in German, 162 p.

Juceviciene A, Zygutiene M, Leinikki P, Brummer-Korvenkontio H, Salminen M, Han X, Vapalahti O (2005) Tick-borne encephalitis virus infections in Lithuanian domestic animals and ticks. Scand J Infect Dis 37(10):742–746, DOI 10.1080/00365540510012134

Katargina O, Russakova S, Geller J, Kondrusik M, Zajkowska J, Zygutiene M, Bormane A, Trofimova J,

Golovljova I (2013) Detection and characterization of tick-borne encephalitis virus in Baltic countries and Eastern Poland. PLoS ONE 8(5):e61374, DOI 10.1371/journal.pone.0061374

Kiffner C, Vor T, Hagedorn P, Niedrig M, Rühe F (2011) Determinants of tick-borne encephalitis virus antibody presence in roe deer (*Capreolus capreolus*) sera. Med Vet Entomol 26(1):18–25, DOI 10.1111/j.1365-2915.2011.00961.x

Kjelland V, Paulsen KM, Rollum R, Jenkins A, Stuen S, Soleng A, Edgar KS, Lindstedt HH, Vaino K, Gibory M, Andreassen ÅK (2018) Tick-borne encephalitis virus, *Borrelia burgdorferi* sensu lato, *Borrelia miyamotoi*, *Anaplasma phagocytophilum* and *Candidatus* Neoehrlichia mikurensis in *Ixodes ricinus* ticks collected from recreational islands in southern Norway. Ticks Tick Borne Dis 9(5):1098–1102, DOI 10.1016/j.ttbdis.2018.04.005

Klaus C, Hoffmann B, Beer M, Müller W, Stark B, Bader W, Stiasny K, Heinz FX, Süss J (2010a) Seroprevalence of tick-borne encephalitis (TBE) in naturally exposed monkeys (*Macaca sylvanus*) and sheep and prevalence of TBE virus in ticks in a TBE endemic area in Germany. Ticks Tick Borne Dis 1(3):141–144, DOI 10.1016/j.ttbdis.2010.06.001

Klaus C, Hoffmann B, Hering U, Mielke B, Sachse K, Beer M, Süss J (2010b) Tick-borne encephalitis (TBE) virus prevalence and virus genome characterization in field-collected ticks (*Ixodes ricinus*) from risk, non-risk and former risk areas of TBE, and in ticks removed from humans in Germany. Clin Microbiol Infect 16(3):238–244, DOI 10.1111/j.1469-0691.2009.02764.x

Klaus C, Beer M, Saier R, Schau U, Moog U, Hoffmann B, Diller R, Süss J (2012) Goats and sheep as sentinels for tick-borne encephalitis (TBE) virus – Epidemiological studies in areas endemic and non-endemic for TBE virus in Germany. Ticks Tick Borne Dis 3(1):27–37, DOI 10.1016/j.ttbdis.2011.09.011

Klaus C, Hörügel U, Hoffmann B, Beer M (2013) Tick-borne encephalitis virus (TBEV) infection in horses: clinical and laboratory findings and epidemiological investigations. Vet Microbiol 163(3–4):368–372, DOI 10.1016/j.vetmic.2012.12.041

Knap N, Korva M, Dolinšek V, Sekirnik M, Trilar T, Avšič-Županc T (2012) Patterns of tick-borne encephalitis virus infection in rodents in Slovenia. Vector Borne Zoonotic Dis 12(3):236–242, DOI 10.1089/vbz.2011.0728

Kowalewska A, Siwińska A, Gut W (1997) [Charakterystyka odpowiedzi immunologicznej dla wirusa kleszczowego zapalenia mózgu w nowych ogniskach zachorowań w 1993 i 1995 roku]. Medycyna ogólna 3:283–289, cited after Pancer K, Gut W (2019) TBE in Poland. In: Dobler G, Erber W, Bröker M, Schmitt HJ(eds) The TBE book, 2nd edn, Global Health Press Pte Ltd, chap 12b, pp 297,Äì304, DOI10.33442/978-981-14-0914-1_12b-25

Kožuch O, Grešíková M, Nosek J, Lichard M, Sekeyová M (1967) The role of small rodents and hedgehogs in a natural focus of tick-borne encephalitis. Bull World Health Organ 36(Suppl. 1):61–66

Krivanec K, Kopecký J, Tomková E, Grubhoffer L (1988) Isolation of TBE virus from the tick *Ixodes hexagonus*. Folia Parasitol 35(3):273–276

Kunz C, Frisch W, Hofmann H, Radda A, Pretzmann G (1967) Follow-up studies of tick-borne encephalitis (ecological, epidemiological and experimental labratory studies). Final technical report for the European Research Office, contract number 91-591-EUC-4031, Institute for Hygiene, University of Vienna, 66 p.

Kunz C, Aspöck H, Frisch W, Hofmann H, Radda A (1969) Studies on tick-borne encephalitis and other arthropod-borne virus diseases. Final technical report for the European Research Office, contract number JA37-68-C-1422, Institute for Hygiene, University of Vienna, 88 p.

Kupča AM, Essbauer S, Zoeller G, de Mendonça PG, Brey R, Rinder M, Pfister K, Spiegel M, Doerrbecker B, Pfeffer M, Dobler G (2010) Isolation and molecular characterization of a tick-borne

encephalitis virus strain from a new tick-borne encephalitis focus with severe cases in Bavaria, Germany. Ticks Tick Borne Dis 1(1):44–51, DOI 10.1016/j.ttbdis.2009.11.002

Laaksonen M, Sajanti E, Sormunen JJ, Penttinen R, Hänninen J, Ruohomäki K, Sääksjärvi I, Vesterinen EJ, Vuorinen I, Hytönen J, Klemola T (2017) Crowdsourcing-based nationwide tick collection reveals the distribution of *Ixodes ricinus* and *I. persulcatus* and associated pathogens in Finland. Emerg Microbes Infect 6(1):1–7, DOI 10.1038/emi.2017.17

Labuda M, Elecková E, Licková M, Sabó A (2002) Tick-borne encephalitis virus foci in Slovakia. Int J Med Microbiol 291(Suppl. 33):43–47, DOI 10.1016/S1438-4221(02)80008-X

Lakos A, Bán E, Schneider F, Nagy A, Mezei E (2019) TBE in Hungary. In: Dobler G, Erber W, Bröker M, Schmitt HJ (eds) The TBE book, 2nd edn, Global Health Press Pte Ltd, chap 12b, pp 297–304, DOI 10.33442/978-981-14-0914-1_12b-14

Larsen AL, Kanestrøm A, Bjørland M, Andreassen Å, Soleng A, Vene S, Dudman SG (2014) Detection of specific IgG antibodies in blood donors and tick-borne encephalitis virus in ticks within a non-endemic area in southeast Norway. Scand J Infect Dis 46(3):181–184, DOI 10.3109/00365548.2013.865140

Levanov L, Vera CP, Vapalahti O (2016) Prevalence estimation of tick-borne encephalitis virus (TBEV) antibodies in dogs from Finland using novel dog anti-TBEV IgG MAb-capture and IgG immunofluorescence assays based on recombinant TBEV subviral particles. Ticks Tick Borne Dis 7(5):979–982, DOI 10.1016/j.ttbdis.2016.05.002

Lindblom P, Wilhelmsson P, Fryland L, Sjöwall J, Haglund M, Matussek A, Ernerudh J, Vene S, Nyman D, Andreassen Å, Forsberg P, Lindgren PE (2014) Tick-borne encephalitis virus in ticks detached from humans and follow-up of serological and clinical response. Ticks Tick Borne Dis 5(1):21–28, DOI 10.1016/j.ttbdis.2013.07.009

Lindhe KES, Meldgaard DS, Jensen PM, Houser GA, Berendt M (2009) Prevalence of tick-borne encephalitis virus antibodies in dogs from Denmark. Acta Vet Scand 51(1):56, DOI 10.1186/1751-0147-51-56

Lommano E, Burri C, Maeder G, Guerne M, Bastic V, Patalas E, Gern L (2012) Prevalence and genotyping of tick-borne encephalitis virus in questing *Ixodes ricinus* ticks in a new endemic area in Western Switzerland. J Med Entomol 49(1):156–164, DOI 10.1603/me11044

Makówka A, Gut W, Stefanoff P (2009) [Detection of TBEV RNA in ticks as a tool for valuation of endemic area and sensitivity of TBE surveillance]. Przegl Epidemiol 63:37–380, in Polish

Mehlhorn H, Mehlhorn T, Müller M, Vogt M, Rissland J (2015) Tick survey for prevalent pathogens in peri-urban recreation sites in Saarland and Rhineland-Palatinate (Germany). Parasitol Res 115(3):1167–1172, DOI 10.1007/s00436-015-4852-x

Melik W, Nilsson AS, Johansson M (2007) Detection strategies of tick-borne encephalitis virus in Swedish *Ixodes ricinus* reveal evolutionary characteristics of emerging tick-borne flaviviruses. Arch Virol 152(5):1027–1034, DOI 10.1007/s00705-006-0922-9

Mierzejewska EJ, Pawełczyk A, Radkowski M, Welc-Falęciak R, Bajer A (2015) Pathogens vectored by the tick, *Dermacentor reticulatus*, in endemic regions and zones of expansion in Poland. Parasit Vectors 8:490, DOI 10.1186/s13071-015-1099-4

Müller K (2006) [Untersuchungen zum Vorkommen von Antikörpern gegen das "Tick Borne Encephalitis Virus" (TBEV) bei Pferden im Endemiegebiet Marburg-Biedenkopf]. PhD thesis, Justus-Liebig-University Giessen, in German, 111 p.

Müller W (1970) [Experimentelle Untersuchungen über das Vorkommen von Arboviren in Unterfranken. 1. Teil: Versuche zur Virusisolierung aus Zecken]. Zbl Bakt, I Abt Orig 214(2):145–159, in German

Nosek J, Kožuch O, Grulich I (1970) The structure of tick-borne encephalitis (TBE) foci in Central

Europe. Oecologia 5(1):61–73, DOI 10.1007/bf00345976

Oehme R, Hartelt K, Backe H, Brockmann S, Kimmig P (2002) Foci of tick-borne diseases in Southwest Germany. Int J Med Microbiol 291(Suppl. 33):22–29, DOI 10.1016/s1438-4221(02)80005-4

Paulsen KM, Stuen S, das Neves CG, Suhel F, Gurung D, Soleng A, Stiasny K, Vikse R, Andreassen ÅK, Granquist EG (2018) Tick-borne encephalitis virus in cows and unpasteurized cow milk from Norway. Zoonoses Public Health 66(2):216–222, DOI 10.1111/zph.12554

Perez-Eid C, Hannoun C, Rodhain F (1992) The Alsatian tick-borne encephalitis focus: Presence of the virus among ticks and small mammals. Eur J Epidemiol 8(2):178–186, DOI 10.1007/bf00144797

Petersen A, Rosenstierne MW, Rasmussen M, Fuursted K, Nielsen HV, O'Brien Andersen L, Bødker R, Fomsgaard A (2019) Field samplings of *Ixodes ricinus* ticks from a tick-borne encephalitis virus micro-focus in Northern Zealand, Denmark. Ticks Tick Borne Dis 10(5):1028–1032, DOI 10.1016/j.ttbdis.2019.05.005

Pettersson JHO, Golovljova I, Vene S, Jaenson TGT (2014) Prevalence of tick-borne encephalitis virus in *Ixodes ricinus* ticks in northern Europe with particular reference to Southern Sweden. Parasit Vectors 7(1):102, DOI 10.1186/1756-3305-7-102

Pintér R, Madai M, Vadkerti E, Németh V, Oldal M, Kemenesi G, Dallos B, Gyuranecz M, Kiss G, Bányai K, Jakab F (2013) Identification of tick-borne encephalitis virus in ticks collected in southeastern Hungary. Ticks Tick Borne Dis 4(5):427–431, DOI 10.1016/j.ttbdis.2013.04.008

Pluta S (2011) [Epidemiology of *Coxiella burnetii*, *Rickettsia* spp., TBE-virus and hantavirus in Southern Germany with regards to climate changes]. PhD thesis, University of Hohenheim, Germany, in German, 255 p.

Radda A, Hofmann H, Kozuch O, Kunz C, Nosek J, Zukrigl K (1971) [Ein Naturherd des Frühsommer-Meningoenzephalitis-Virus bei St. Florian]. Anz Schädlkd Pflanzenschutz Umweltschutz 44(6):96–96, DOI 10.1007/BF02040652, in German

Ramelow C, Süss J, Berndt D, Roggendorf M, Schreier E (1993) Detection of tick-borne encephalitis virus RNA in ticks (*Ixodes ricinus*) by the polymerase chain reaction. J Virol Methods 45(1):115–119, DOI 10.1016/0166-0934(93)90145-h

Rieger MA, Nübling M, Müller W, Hasselhorn HM, Hofmann F (1999) Foxes as indicators for TBE endemicity—a comparative serological investigation. Zbl Bakteriol 289(5–7):610–618, DOI 10.1016/s0934-8840(99)80017-6

Rieille N, Bressanelli S, Freire CCM, Arcioni S, Gern L, Péter O, Voordouw MJ (2014) Prevalence and phylogenetic analysis of tick-borne encephalitis virus (TBEV) in field-collected ticks (*Ixodes ricinus*) in southern Switzerland. Parasit Vectors 7(1):443, DOI 10.1186/1756-3305-7-443

Rieille N, Klaus C, Hoffmann D, Péter O, Voordouw MJ (2017) Goats as sentinel hosts for the detection of tick-borne encephalitis risk areas in the Canton of Valais, Switzerland. BMC Vet Res 13:217, DOI 10.1186/s12917-017-1136-y

Rijks JM, Montizaan MG, Bakker N, de Vries A, Van Gucht S, Swaan C, van den Broek J, Gröne A, Sprong H (2019) Tick-borne encephalitis virus antibodies in roe deer, the Netherlands. Emerg Infect Dis 25(2):342–345, DOI 10.3201/eid2502.181386

Roelandt S, Heyman P, De Filette M, Vene S, Van der Stede Y, Caij AB, Tavernier P, Dobly A, De Bosschere H, Vyt P, Meersschaert C, Roels S (2011) Tick-borne encephalitis virus seropositive dog detected in Belgium: screening of the canine population as sentinels for public health. Vector Borne Zoonotic Dis 11(10):1371–1376, DOI 10.1089/vbz.2011.0647

Roelandt S, Suin V, Riocreux F, Lamoral S, Van der Heyden S, Van der Stede Y, Lambrecht B, Caij B, Brochier B, Roels S, Van Gucht S (2014) Autochthonous tick-borne encephalitis virus-seropositive

cattle in Belgium: a risk-based targeted serological survey. Vector Borne Zoonotic Dis 14(9):640–647, DOI 10.1089/vbz.2014.1576

Roelandt S, Suin V, Van der Stede Y, Lamoral S, Marche S, Tignon M, Saiz JC, Escribano-Romero E, Casaer J, Brochier B, Van Gucht S, Roels S, Vervaeke M (2016) First TBEV serological screening in Flemish wild boar. Infect Ecol Epidemiol 6(1):31099, DOI 10.3402/iee.v6.31099

Rosà R, Tagliapietra V, Manica M, Arnoldi D, Hauffe HC, Rossi C, Rosso F, Henttonen, Rizzoli A (2019) Changes in host densities and co-feeding pattern efficiently predict tick-borne encephalitis hazard in an endemic focus in northern Italy. Int J Parasitol 49(10):779–787, DOI 10.1016/j.ijpara.2019.05.006

Salat J, Mihalca AD, Mihaiu M, Modrý D, Ruzek D (2017) Tick-borne encephalitis in sheep, Romania. Emerg Infect Dis 23(12):2065–2067, DOI 10.3201/eid2312.170166

Sidorenko M, Radzievskaja J, Rosef O, Paulauskas A (2018) Investigation of the tick-borne encephalitis virus in Norway. Biologija 64(2):172–178, DOI 10.6001/biologija.v64i2.3741

Šikutová S, Hornok S, Hubálek Z, Doležálková I, Juřicová Z, Rudolf I (2009) Serological survey of domestic animals for tick-borne encephalitis and Bhanja viruses in northeastern Hungary. Vet Microbiol 135(3–4):267–271, DOI 10.1016/j.vetmic.2008.09.082

Širmarová J, Tichá L, Golovchenko M, Salát J, Grubhoffer L, Rudenko N, Nowotny N, Růžek D (2014) Seroprevalence of *Borrelia burgdorferi* sensu lato and tick-borne encephalitis virus in zoo animal species in the Czech Republic. Ticks Tick Borne Dis 5(5):523–527, DOI 10.1016/j.ttbdis.2014.03.008

Skarphédinsson S, Jensen PM, Kristiansen K (2005) Survey of tickborne infections in Denmark. Emerg Infect Dis 11(7):1055–1061, DOI 10.3201/eid1107.041265

Skuballa JD (2011) The role of the European hedgehog (*Erinaceus europaeus*) in the epidemiology of tick-borne diseases (*Borrelia* spp., *Anaplasma* spp., *Rickettsia* spp. and TBE-virus). PhD thesis, Karlsruhe Institute of Technology, Germany, in German, 242 p.

Smura T, Tonteri E, Jääskeläinen A, von Troil G, Kuivanen S, Huitu O, Kareinen L, Uusitalo J, Uusitalo R, Hannila-Handelberg T, Voutilainen L, Nikkari S, Sironen T, Sane J, Castrén J, Vapalahti O (2019) Recent establishment of tick-borne encephalitis foci with distinct viral lineages in the Helsinki area, Finland. Emerg Microbes Infect 8(1):675–683, DOI 10.1080/22221751.2019.1612279

Sormunen JJ, Klemola T, Hänninen J, Mäkelä S, Vuorinen I, Penttinen R, Sääksjärvi IE, Vesterinen EJ (2018) The importance of study duration and spatial scale in pathogen detection—evidence from a tick-infested island. Emerg Microbes Infect 7(1):1–11, DOI 10.1038/s41426-018-0188-9

Stefanoff P, Siennicka J, Kaba J, Nowicki M, Ferenczi E, Gut W (2008) Identification of new endemic tick-borne encephalitis foci in Poland – a pilot seroprevalence study in selected regions. Int J Med Microbiol 298(Suppl. 1):102–107, DOI 10.1016/j.ijmm.2008.04.002

Stefanoff P, Pfeffer M, Hellenbrand W, Rogalska J, Rühe F, Makówka A, Michalik J, Wodecka B, Rymaszewska A, Kiewra D, Baumann-Popczyk A, Dobler G (2012) Virus detection in questing ticks is not a sensitive indicator for risk assessment of tick-borne encephalitis in humans. Zoonoses Public Health 60(3):215–226, DOI 10.1111/j.1863-2378.2012.01517.x

Süss J, Béziat P, Ramelow C, Kahl O (1997) Tick-borne encephalitis virus (TBEV)-specific RT-PCR for characterization of natural foci of TBE and for other applications. Zbl Bakteriol 286(1):125–138, DOI 10.1016/s0934-8840(97)80084-9

Süss J, Klaus C, Diller R, Schrader C, Wohanka N, Abel U (2006) TBE incidence versus virus prevalence and increased prevalence of the TBE virus in *Ixodes ricinus* removed from humans. Int J Med Microbiol 296(Suppl. 40):63–68, DOI 10.1016/j.ijmm.2005.12.005

Tavernier P, Sys SU, De Clercq K, De Leeuw I, Caij AB, De Baere M, De Regge N, Fretin D, Roupie V, Govaerts M, Heyman P, Vanrompay D, Yin L, Kalmar I, Suin V, Brochier B, Dobly A, De Craeye

S, Roelandt S, Goossens E, Roels S (2015) Serologic screening for 13 infectious agents in roe deer (*Capreolus capreolus*) in Flanders. Infect Ecol Epidemiol 5(1):29862, DOI 10.3402/iee.v5.29862

Tonteri E, Jokelainen P, Matala J, Pusenius J, Vapalahti O (2016) Serological evidence of tick-borne encephalitis virus infection in moose and deer in Finland: sentinels for virus circulation. Parasit Vectors 9(1):54, DOI 10.1186/s13071-016-1335-6

Traavik T (1973) Serological investigations indicating the existence of tick-borne encephalitis virus foci along the Norwegian coast. APMIS 81B(1):138–142, DOI 10.1111/j.1699-0463.1973.tb02197.x

Traavik T, Mehl R, Wiger R (1978) The first tick-borne encephalits virus isolates from Norway. APMIS 86B(1–6):253–256, DOI 10.1111/j.1699-0463.1978.tb00040.x

von Zeipel G (1959) Isolation of viruses of the Russian spring-summer encephalitis-louping ill group from Swedish ticks and from a human case of meningoencephalitis. Arch Gesamte Virusforsch 9(4):460–469, DOI 10.1007/bf01242853

von Zeipel G, Svedmyr A, Zetterberg B (1959) The geographical distribution in Sweden of viruses belonging to the Russian spring-summer-louping ill group. Arch Gesamte Virusforsch 9(4):449–459, DOI 10.1007/bf01242852

Wandeler A, Steck F, Fankhauser R, Kammermann B, Grešíková M, Blašcovič D (1972) Isolierung des Virus der zentraleuropäischen Zeckenenzephalitis in der Schweiz. Path Microbiol 38(4):258–270, DOI 10.1159/000162416

Weidmann M, Růžek D, Křivanec K, Zöller G, Essbauer S, Pfeffer M, de A Zanotto PM, Hufert FT, Dobler G (2011) Relation of genetic phylogeny and geographical distance of tick-borne encephalitis virus in central Europe. J Gen Virol 92(8):1906–1916, DOI 10.1099/vir.0.032417-0

Weissenböck H, Suchy A, Holzmann H (1998) Tick-borne encephalitis in dogs: neuropathological findings and distribution of antigen. Acta Neuropathol 95(4):361–366, DOI 10.1007/s004010050811

Wicki R, Sauter P, Mettler C, Natsch A, Enzler T, Pusterla N, Kuhnert P, Egli G, Bernasconi M, Lienhard R, Lutz H, Leutenegger CM (2000) Swiss army survey in Switzerland to determine the prevalence of *Francisella tularensis*, members of the *Ehrlichia phagocytophila* genogroup, *Borrelia burgdorferi* sensu lato, and tick-borne encephalitis virus in ticks. Eur J Clin Microbiol Infect Dis 19(6):427–432, DOI 10.1007/s100960000283

Wójcik-Fatla A, Cisak E, Zając V, Zwoliński J, Dutkiewicz J (2011) Prevalence of tick-borne encephalitis virus in *Ixodes ricinus* and *Dermacentor reticulatus* ticks collected from the Lublin region (eastern Poland). Ticks Tick Borne Dis 2(1):16–19, DOI 10.1016/j.ttbdis.2010.10.001

Ytrehus B, Vainio K, Dudman SG, Gilray J, Willoughby K (2013) Tick-borne encephalitis virus and Louping-ill virus may co-circulate in Southern Norway. Vector Borne Zoonotic Dis 13(10):762–768, DOI 10.1089/vbz.2012.1023

Zając V, Wójcik-Fatla A, Sawczyn A, Cisak E, Sroka J, Kloc A, Zając Z, Buczek A, Dutkiewicz J, Bartosik K (2017) Prevalence of infections and co-infections with 6 pathogens in *Dermacentor reticulatus* ticks collected in eastern Poland. Ann Agric Environ Med 24(1):26–32, DOI 10.5604/12321966.1233893

Zöldi V, Papp T, Rigó K, Farkas J, Egyed L (2014) A 4-year study of a natural tick-borne encephalitis virus focus in Hungary, 2010–2013. EcoHealth 12(1):174–182, DOI 10.1007/s10393-014-0969-0
